# Recent Advances in Synthetic, Industrial and Biological Applications of Violacein and Its Heterologous Production

**DOI:** 10.4014/jmb.2107.07045

**Published:** 2021-09-28

**Authors:** Aqsa Ahmed, Abdullah Ahmad, Renhan Li, Waleed AL-Ansi, Momal Fatima, Bilal Sajid Mushtaq, Samra Basharat, Ye Li, Zhonghu Bai

**Affiliations:** 1 School of Biotechnology, Jiangnan University, Wuxi 214122, P.R. China; 2 National Engineering Laboratory for Cereal Fermentation Technology, Jiangnan University, Wuxi 214122, P.R. China; 3 Department of Industrial Biotechnology, Atta-Ur-Rahman School of Applied Biosciences, National University of Science and Technology, Islamabad 44000, Pakistan; 4 School of Food Science and Technology, State Key Laboratory of Food Science and Technology, Jiangnan University, 1800 Lihu Avenue, Wuxi 214122, P.R. China; 5 Department of Food Science and Technology, Faculty of Agriculture, Sana’a University, Sana’a, 725, Yemen; 6 Department of Industrial Biotechnology, National Institute of Biotechnology and Genetic Engineering (NIBGE), Faisalabad 38000, Pakistan

**Keywords:** Violacein, heterologous production, industrial applications, biological applications, synthetic biology, metabolic engineering.

## Abstract

Violacein, a purple pigment first isolated from a gram-negative coccobacillus *Chromobacterium violaceum*, has gained extensive research interest in recent years due to its huge potential in the pharmaceutic area and industry. In this review, we summarize the latest research advances concerning this pigment, which include (1) fundamental studies of its biosynthetic pathway, (2) production of violacein by native producers, apart from *C. violaceum*, (3) metabolic engineering for improved production in heterologous hosts such as *Escherichia coli*, *Citrobacter freundii*, *Corynebacterium glutamicum*, and *Yarrowia lipolytica*, (4) biological/pharmaceutical and industrial properties, (5) and applications in synthetic biology. Due to the intrinsic properties of violacein and the intermediates during its biosynthesis, the prospective research has huge potential to move this pigment into real clinical and industrial applications.

## Introduction

**Fig. 1:**
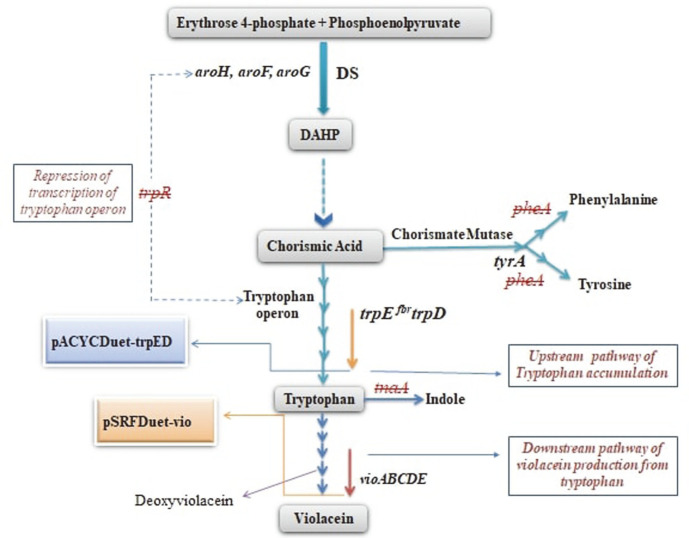
Metabolic engineering for the accumulation of tryptophan. The red cross indicates targeted genes to be knocked out that are responsible for transcription of the tryptophan operon (*trpR*) , competition with chorismate to create other aromatic amino acids (*pheA*) , and tryptophan degradation (*tnaA*). 3-deoxy-D-arabinoheptulosonate 7-phosphate synthase (DS), 3-deoxy-D-arabinoheptulosonate 7-phosphate (DAHP).

Violacein is a purple-colored, natural indole derivative that is biosynthesized by the condensation of two tryptophan molecules in several bacterial genera in response to quorum-sensing signals [1]. Its chemical structure consists of three structural units, a 5-hydroxy indole, an oxindole, and a 2-pyrrolidone [2]. The biosynthetic pathway of violacein from L-tryptophan has been clarified, involving five enzymes (VioA, B, E, D, and C, sequentially), which are encoded in the *vioABCDE* operon [3] as shown in Fig. 1.

 Violacein was first isolated from *C. violaceum*, the most studied bacteria particularly for its potential for violacein production [4]. Later, several other bacterial genera that also produce violacein were isolated and characterized, including strains of psychrotrophic bacteria [5], *Janthinobacterium lividum* [2], *Duganella* [6], *Collimonas* [7], *Massilia* [8], *Pseudoalteromonas* [9] and *Antarctic Iodobacter* [10].

Apart from being a quorum-sensing metabolite, violacein tends to have a broad range of biological activities, including anti-tumoral [11], bacteriostatic and antibiotic potential [12, 13], antifungal [14], anti-protozoan [15], anti-cancer [16, 17], and antiviral properties [18]. Violacein not only possesses the potential of working alone as an antibiotic, but it also has the potential of acting synergistically with other antibiotics [10]. Meanwhile, the by-product deoxyviolacein (synthesized from L-tryptophan by VioA, B, E, and C, sequentially), shows stronger antifungal properties than antimicrobial properties as compared to violacein [19]. Moreover, violacein is of immense industrial importance and has applications in cosmetics, textiles, agriculture, and drug discovery [18].

These interesting properties drive researchers to improve violacein production in the native producers (such as *C. violaceum* and *Duganella* sp. B2) by optimization of culture conditions [19], but the extensive prospects of violacein in industry demand more yield than is being achieved by natural producers [20]. To overcome the issues of low productivity and bacterial pathogenicity of the native producers, the *vioABCDE* gene cluster has been cloned and expressed in heterologous hosts, such as *E. coli*, *Citrobacter freundii*, *Corynebacterium glutamicum*, and *Y. lipolytica*. Through comprehensive optimization of expression of *vioABCDE*, primary metabolism to enhance L-tryptophan supply, as well as culture conditions, violacein production has improved significantly [20-24].

In recent years, the vivid hues of violacein and the intermediates in the violacein biosynthetic pathway have also been utilized by synthetic biologists to test their designs leading to potential research applications of violacein such as evaluation of translocation efficiency of peroxisomal tags, efficiency of golden gate assembly, and the development of biosensors and combinatorial expression libraries, etc. [25-27], implying the potential for more applications of this pathway in synthetic biology through delicate design.

In this review, we summarized the latest knowledge of how violacein is synthesized, engineered production in various natural and recombinant producers, along with its pharmacological activities and industrial potentials as well as applications in synthetic biology. A recent article also focuses on this pigment [28], but our review differs in its detailed structure and synthetic biology perspective that can pave ways for further research in this particular area.

## Biochemical Aspects of Violacein Biosynthesis

**Fig. 2:**
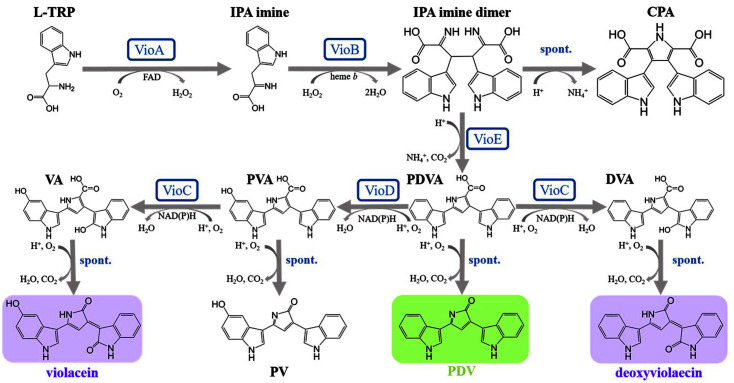
Biosynthesis of violacein.

The violacein biosynthetic pathway has been well studied. As illustrated in Fig. 2, it involves five enzymes, VioA, B, C, D, and E [29], (1) L-tryptophan is converted to indole 3-pyruvic acid (IPA) imine by the flavin-dependent tryptophan-2 monooxygenase enzyme VioA. (2) IPA imine is dimerized to a transient imine dimer, which is catalyzed by VioB. VioB has catalase activity with heme *b* as cofactor. (3) VioE converts the imine dimer to protoviolaceinic acid (PDVA) by 1,2-shift of an indole ring. In the absence of VioE, the unstable imine dimer spontaneously converts to chromopyrrolic acid (CPA). (4) PDVA is then converted into protoviolaceinic acid (PVA) by an NADP-dependent oxygenase VioD by adding one hydroxyl group on the C5 position of one indole ring. (5) PVA is converted to violaceinic acid (VA) by another NADP-dependent oxygenase VioC, by adding one hydroxyl group on the C2 position of the other indole ring, followed by the formation of the final product violacein via spontaneous oxidative decarboxylation. The promiscuity of VioC enables its use of PDVA as the substrate and results in the formation of deoxyviolacein, the major by-product. Thus, typically a mixture of violacein and deoxyviolacein is obtained when the whole *vioABCDE* operon is expressed, which is termed as “crude violacein” [3, 29]. Upon *vioD* deletion, pure deoxyviolacein can be synthesized [30].

**Fig. 3:**
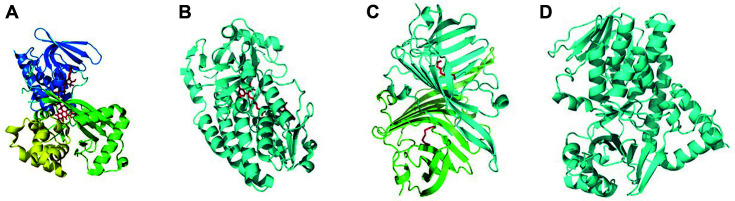
Crystal structures of (a) VioA (PDBID:5G3T): FAD-binding domain (blue), substrate-binding domain (green), helical domain (yellow), FAD (red), (b) VioD and FAD (red) (PDBID: 3C4A), and (c) VioE dimer and PEG (red) (PDBID: 3BMZ). (d) VioC homogly model based on kynurenine 3-monooxygenase from *Rattus norvegicus* (PDBID: 6LKE).

So far, the crystal structures of VioA, VioD, and VioE have been elucidated [31, 32], while the structures of VioB and VioC are still missing. The most well-characterized L-tryptophan oxidase VioA is a “loosely associated” homodimer with each monomer composed of a FAD-binding domain, a substrate-binding domain, and a helical domain (Fig. 3A). Multiple versions of its structures have been reported (PDB IDs: 5G3S, 5G3T, 5G3U, 5ZBC, 5ZBD, 6ESD, 6G2P, 6FW7-9, 6FWA). Füller *et al*. used the samarium nitrate-derived crystals to resolve the phase problem and obtained the structure of VioA from *C. violaceum*, in complex with FAD (5G3S and 5G3T), as well as FAD and its inhibitor IEA (5G3U) [32]. In another study, Lai *et al*. elucidated the structure of VioA in complex with FAD and its native substrate L-tryptophan (6G2P) [33]. What’s more, they also obtained a collection of VioA structures with different substrate analogs, including 4-fluoro-DL-tryptophan (6FW7), 5-methyl-DL-tryptophan (6FW8), 6-fluoro-DL-tryptophan (6FW9), and 7-methyl-DL-tryptophan (6FWA). In another study, Yamaguchi *et al*. constructed the VioA (C395A) variant to improve its thermal stability [34]. The VioA (C395A) structure with FAD and L-tryptophan (5ZBD), as well as the Se-Met modified version with FAD (5ZBC), enabled analysis of the improved stability and substrate specificity from structural insights. The co-crystalized structure of the hydroxylase VioD with FAD was reported more than a decade ago (PDB ID: 3C4A), but its structural characterization remains to be elucidated (Fig. 3B). As for the VioE, its crystal structures (PDB ID: 2ZF3, 2ZF4, and 3BMZ) reveal that it shares a core fold of lipoprotein transporters. The structural and mutagenesis studies indicated its role as a catalytic chaperone that segregates the hydrophobic substrate from its surroundings so as to initiate the rearrangement reaction [31]. Currently, the crystal structures of VioB and VioC are still not available. In this work, we searched the homology models with known crystal structures for VioB and VioC, using the Swiss-Model online server. As for VioB, the template with highest identity is a ferritin-like protein from *Caulobacter vibriodes* (PDB ID: 3hl1), with sequence identity of 19.85% and a GMQE (Global Model Quality Estimation) value of only 0.15. Such low modeling scores along with largely unknown catalytic properties would account for the structural biologists’ lack of interest in elucidating its structure. In constrast, the monooxygenase VioC shows sequence identity of 27.25% and a GMQE value of 0.70 with the kynurenine 3-monooxygenase from *Rattus norvegicus* (PDB ID: 6LKE), which allows us to build up a VioC homology model (Fig. 3D). To summarize, the structural information described above not only reveals partial insights into how violacein is biosynthesized from the molecular level, but also provides opportunities to engineer the promiscuity to allow production of non-natural violacein derivatives with modified indole rings.

## Biological Activities of Violacein/Deoxyviolacein

Violacein has proved itself of commercial importance due to a range of biological and industrial activities. Many studies have shown that violacein exhibits multiple biological properties, *e.g.*, anti-bacterial [13], anti-fungal [14], anti-cancer [16, 17], antiviral properties [18], etc. In the following section, we review the latest advances on studies of its biological properties in detail, along with a brief summary in Table 3.

### Anti-Microbial Activity

Violacein exhibits broad-spectrum anti-microbial activities. Violacein has been checked alone for its antibacterial activity as well as in combination with other antibacterial drugs and agents extensively against *Staphylococcus aureus*. For example, violacein shows antibiotic effect on *S. aureus*, which is the causative agents of bovine mastitis [35]. The minimal inhibitory concentration (MIC) for *S. aureus* was established to be in the range of 6.25-25 μmol/l. When violacein was used synergistically with penicillin, the MIC decreased to 3.12 μmol/l, but did not decrease with streptomycin (6.25-40 μmol/l) [36]. The effects of violacein in relation to *S. aureus* and *Salmonella enterica* were studied in combination with kanamycin and azithromycin. The MIC values were found to be 16.6 μmol/l for these species. Similarly, MIC values were adjusted to be 45.46 μmol/l, 53.91 μmol/l, and 58.28 μmol/l for *K. pneumoniae*, *P. aeruginosa*, and *V. cholerae*, respectively. Combining violacein with cefadroxil and gentamycin further decreased the MIC value to 2.91 μmol/l [37]. In another study, S. aureus ATCC 29213 and methicillin-resistant *S. aureus* ATCC 43300 were treated with vancomycin and violacein at their MIC values, and it was found that violacein showed four times the activity of vancomycin, depicting a much greater leakage of cellular ATP [13]. Violacein has also been tested for its antibacterial potential in combination with nanoparticles to facilitate their delivery and for the synergistic antibacterial effect. Violacein in free form and loaded in poly-(D, L-lactide-co-glycoside) nanoparticles were both tested and showed antibacterial effect against *S. aureus* ATCC 25923. The loaded violacein exhibited three times more antibacterial activity as compared to free violacein [38]. In another study, violacein-capped silver nanoparticles were tested against methicillin-resistant *S. aureus* (MRSA), *P. aeruginosa*, and *E. coli* and the resultant antibacterial effect was much greater as compared to starch capped silver nanoparticles [39]. Violacein when combined with silk fabric resulted in anti-bacterial properties of the composite, and it was further integrated with silver nanoparticles (SNPs) to form functional silk composites (FSCs). To test the antibacterial effects of these antibacterial fiber composites, violacein-integrated silk fabric was exposed to *S. aureus* with a bacterial population reduction of 81.25%. On the other hand, FSCs with integrated silver nanoparticles resulted in a much greater and broad range reduction of *S. aureus* (99.98%), *E. coli* (99.90%), and *Candida Albicans* (99.85%) [40].

Apart from *S. aureus*, violacein has also been tested against many other bacterial species. Violacein extracted from *Janinthobacterium* sp. was tested against several strains of gram-negative multidrug-resistant (MDR) bacteria. MDR organisms are a class of microorganisms that show resistance to at least one antimicrobial drug in three or more antimicrobial classes. The MIC values were found to be 1.4-46.6 nmol/l, and it significantly inhibited *P. aeruginosa* and *S. marcescens* [26]. Moreover, a strong antifungal activity against *Rhizoctonia solani* was demonstrated by deoxyviolacein at concentrations beyond 6.1 mmol/l, while oxyviolacein showed activity against a vegetable crop pathogen called *Phytophtora capsica* along with antifungal activity against *Botrytis cinerea*, *Verticillium dahliae*, and *Fusarium oxysporum* at 1.5 mmol/l [41, 42]. In another study, violacein obtained from *Duganella violaceinigra* showed antibacterial activity against *S. aureus*, even the MDR strains at an MIC value of 15 μmol/l [43]. Violacein was also studied to check its effect on the mammalian gut microbiome showing that different concentrations of violacein affect the gut microflora. At low concentrations (145 μmol/l), *Bacilli* and *Clostridia* remained to be the most dominant group, whereas, at high concentrations (1457 μmol/l) of violacein, *Clostridia* and *Actinobacteria* prevailed as the most surviving groups, whereas the rest of the microbiota were modulated [44]. Considering the hydrophobic nature of violacein, extracellular membrane vesicles (MVs) of *C. violaceum* were utilized for solubilizing and transferring violacein to the microorganisms. The solubility of violacein in MVs was increased up to 1,740 folds, which killed 90% of *S. aureus* (3.1 mg/l) in 6 h [45].

Violacein also exhibits antifungal properties against many fungal pathogens like *Candida albicans*, *Candida tropicalis*, *Aspergillus flavus*, *Fusarium oxysporum*, *Penicillium expansum*, and *Rhizoctonia solani* [46]. In a study conducted in 2018, violacein obtained from cutaneous bacteria of amphibians showed antifungal activities against *Batrachochytrium dendrobatidis* and *Batrachochytrium salamandrivorans* [47]. Apart from these examples, violacein has shown antifungal activities against *Botrytis cinerea*, and *Colletotrichum acutatum* [48], *Colletotrichum glycines*, *Colletotrichum orbiculare*, *Gibberella zeae*, *Phytophthora capsica*, and *Verticillium dahlia* [41], etc. Violacein has also shown weak inhibitory effects against viruses like Simian rotavirus SA11, HSV-1, and Poliovirus type 2 [49].

### Anti-Parasitic Activity

Violacein demonstrated its anti-malarial potential by showing significant results against *Plasmodium falciparum* and *Plasmodium chabaudi* both in vitro and in vivo in mice [50]. In another study, violacein exhibited mild cytotoxicity against *Trypanosoma brucei gambiense* in vitro with an IC_50_ value of 0.1 μmol/l [51].

Violacein has also shown anti-leishmanial activity against *Leishmania amazonensis* at an EC_50_/24 h value of 4.3 ± 1.15 μmol/l. Although pentamidine showed better results against the disease, violacein is comparatively less toxic [52]. Violacein also showed considerable anti-nematodal activity against *C. elegans* (LC_50_ > 30 μmol/l) (LC stands for lethal concentration; the concentration of a material which kills 50% of test animals after single exposure) [53]. A glycodiversified violacein variant 5′-O-glucoside produced in *E. coli* expressing the *vioABCDE* cluster, as well as a glucosyl transferase gene (yjiC), was demonstrated. This particular variant exhibited anti-nematodal activity against *Bursaphelenchus xylophilus*, which is responsible for pine wilt disease [54].

### Immunomodulatory Activity

In the last decade, studies have been carried out on the immunomodulatory, analgesic, and antipyretic activities of violacein. In a detailed experiment consisting of various steps, violacein was found to be involved in immunosuppressive, antinociceptive, analgesic, and antipyretic effects [55]. Violacein reduced gastrointestinal inflammation, possibly through COX-1 mediated pathways in ulcer rat models [56], while another study found that violacein can have immunomodulatory effects by regulating cytokine production when injected directly into the intraperitoneal cavity: it decreased the expression of IL-6 and TNF but increased the expression of IL-1 [57]. In contrary to this, a study reported previously that the TNF expression was increased in HL60, and TNF receptor 1 signaling was also triggered when this cell line was exposed to violacein [58]. In MCF-7 cells, violcein has been shown to boost TNF expression and upregulate the p53-dependent mitochondrial pathway [17] , while TNF expression in RAW 264.7 and ANA-1 cells was also stimulated upon violacein administration [59]. These variations could be attributable to the different experimental techniques used, such as in vitro or in vivo methods of performing experiments and also the type of cells used [14].

### Anti-Cancer Properties

Violacein has become a product of interest due to its anti-cancer properties [60]. Chemotherapeutics usually have a limitation of non-specific toxicity but violacein has exhibited apoptosis induction and anti-tumoral effect against certain specific tumor cell lines, such as cancer cell lines [11, 61]. Much of the work done previously on the antitumor activities of violacein has already been reviewed [4, 18].

In an attempt to understand the mechanism of action of violacein and the reason behind its cytotoxicity on cancer cell lines, its effect on protein kinases was studied. For this purpose, phosphorylation experiments were carried out on classical-type protein kinase C (PKC) and other atypical and novel protein kinases. The results demonstrated a strong inhibitory effect on the catalytic subunits of protein kinase A (PKA) and PKC. This led to a better understanding of violacein toxicity by its targeting of catalytic subunits of protein kinases [62]. In another study, it was found that hypoxia resulted in an increase in the violacein anti tumor activity in MCF7 cell lines, HCT116 colon cancer cells, HN5 head and neck squamous cell carcinoma, and HT29 colon cancer cells [63].

Similarly, Platt *et al*. checked the violacein toxicity against breast cancer cell line MCF7 and concluded that it contributed to downregulate the CXCL12/CXCR4 interaction in these cells [64]. In another study, 1 μmol/L of violacein was found to inhibit lung cancer (A549), glioblastoma (U87), and breast cancer (MCF7) cell lines. In a similar study, violacein was found to inhibit MCF7 and human fetal lung fibroblast (MRC-5) cell lines via mitochondrial dysfunction thus eventually leading to cell death [65].

Recently, violacein was extracted from an Antarctic bacterial isolate and after optimizing its production by changing media temperature and composition it was tested for its anti-proliferative properties against HeLa (cervix cell carcinoma) cell lines. The results indicated that treatment of violacein rendered them sensitive to cisplatin, an anti tumor drug. This study indicates the potential of using combinatorial strategies by combining violacein with cisplatin or other anti tumor drugs for better results [66]. In another study, a violacein carrier was designed for better delivery of violacein to cancer cells. Being hydrophobic, violacein was emulsified along with polyoxyethylene sorbitan monolaurate in order to increase its stability in an aqueous environment. It was further encapsulated with Pectin-Gelatin resulting in a microsphere. The carrier was tested on HTC-116 colon cancer cell lines and violacein emulsion drastically reduced their viability, however, the pectin-gelatin coating proved to be a limitation since it attenuated violacein’s effect [67].

Mitochondrial dysfunction is the main reason for many cancer pathologies. TFAM is a transcription factor A of mitochondria that plays a role in the synthesis of various mitochondrial proteins leading to malignancy. Research is being done on making a dyad drug system, comprising violacein and silver nanoparticles, that has the ability to structurally bind and inhibit TFAM at the interface of TFAM-DNA complex during replication and contribute towards hindering the majority of pathways causing cancer proliferation. Molecular docking studies of TFAM-DNA with violacein and silver nanoparticles led to -8.836 kcal/mol binding energy and 1.51 μmol Ki value (inhibition constant). This hypothesis is further strengthened by a good binding score of 9518 of silver nanoparticles in the TFAM’s DNA interacting cavity [68].

### Nephroprotective Activity

The administration of violacein obtained from *C. violaceum* showed nephroprotective activity against heavy metals and gentamicin through an antioxidant property in Wistar rats. Violacein exhibited protective properties on the convoluted proximal tubule as well as the straight proximal tubule. Violacein acted to control the increased amounts of uric acid, urea and creatinine induced by cadmium-chloride, potassium dichromate, and gentamycin [69].

### Anti-Diarrheal and Ulcer-Protective Properties

Using castor oil, magnesium sulphate, and ethanol, the anti-diarrheal and ulcer-protective effects of violacein isolated from *C. violaceum* were examined in Wistar rats. Their intestinal transit was significantly slowed and stomach emptying was delayed; 40 mg/kg of violacein had a higher anti-motility effect than 0.1 mg/kg of atropine. Violacein showed ulcer-protective characteristics against ethanol-induced ulceration, with anti-ulcer activity peaking at 40 mg/kg. Moreover, violacein (40 mg/kg) inhibited castor oil-induced diarrhea in rats by 87.84% [70]. Violacein had a strong gastroprotective effect against indomethacin-induced lesions, but L-NAME and glibenclamide were able to counteract this effect. Pretreatment with violacein reduced apoptosis and gastric microvascular permeability while also restoring cNOS levels to baseline [56].

## Potential Industrial Applications

The extensive research on violacein including its genome, biosynthetic pathway, metabolic intermediates, and other studied attributes could lead to several potential industrial applications of this pigment derived from various bacterial and other sources. Natural pigments were rarely used in various industries (textile, cosmetic, food, and pharmaceuticals) because of their high cost and low yield while synthetic pigments hinder their exploitation due to toxicity and other difficulties. These issues were overcome by Aruldass *et al*. [71]. Also, to facilitate the application of violacein as a food additive, *C. glutamicum* yielded 15.727 mmol/l of violacein when employed as a chassis for violacein production in a 3-L bioreactor [20]. In a recent study, natural colorants including carotenoids, indigo, anthocyanins, and violacein were produced using *E. coli* strains. The enhanced production of rainbow colorants was accomplished by integrated membrane engineering strategies. All seven colors were covered by carotenoids and violacein derivatives because of their health-related beneficial properties in food, cosmetics, and drug industries. The strategic engineering enhanced the rainbow colorant production to 0.539 mmol/l of astaxanthin (red), 0.3832 mmol/l of zeaxanthin (yellow), 0.638 mmol/l of β-carotene (orange), 2.711 mmol/l of prodeoxyviolacein (blue), 34.4 mmol/l of deoxyviolacein (purple), 4.1 mmol/l of proviolacein (green) and 20.09 mmol/l of violacein (navy) [72]. In another study, violacein in combination with ink and glycerol was used to highlight printed words with a good result, thus stressing the need for expanding the industrial applications of violacein [73]. Violacein-enhanced UV absorption in the range 290 to 320 nm showed that its addition to other sunscreen ingredients could increase the sunscreen protection factors (SPF). It is also supplemented with *Aloe vera* leaf extract and *Cucumis sativus*; both having photoprotective activity. The above-mentioned attributes of violacein indicate that it has a potential biotechnological role as a photo protectant [74].

The purple color of violacein is a distinctive feature of this pigment that is exploited by the textile industry. It was used to dye fibrous materials and nylon cloth after being manufactured by culturing *Chromobacterium* and *Janthinobacterium* in a specific media. Likewise, natural (silk, cotton, and wool) and chemical fibers (nylon and vinylon) can also be used. The selected color tone was bluish-purple/light purple [4]. Violacein pigment on the fabrics also exhibited antimicrobial activity against *Rosellinia necatrix*; a phytopathogenic fungus [75]. Violacein obtained from *J. lividum* can be used as a natural dye since it is stable at the pH range of 2-11 and at a temperature of 55°C [76]. In a recent study, violacein from *J. lividum* cultures was effectively used for dying fabrics by eco-friendly approaches giving them antifungal and antibacterial properties. The dyed fabrics were resistant against *Candida albicans*, *C. parapsilosis*, and *C. krusei*, as well as *E. coli*, *Staphylococcus aureus*, and MRSA [77].

Violacein in combination with a lipophilic substance and/or a hydrophilic substance is used in cosmetic formulations along with its derivatives as dyes for skin and hair preparation. It is also used as an antimicrobial agent in cosmetics. The strong antibiotic activity of violacein against *S. aureus* and its antioxidant effect on linoleic acid led to the formulation of a cosmetic lotion [78]. Violacein-capped silver nanoparticles (vAgNPs) also have applications in the cosmetic industry as beneficial skin products like anti-aging creams [39]. Violacein has shown considerable larvicidal and pupicial properties against certain insects, although the mechanism of action of violacein against insects is still unclear. It also proved efficient in controlling anthracnose, stem rot, pythium blight, and damping-off of bean sprouts by inhibiting the growth of pathogenic fungi and parasitic nematodes that are responsible for damaging plants. Insecticides containing violacein and its derivatives have the ability to prevent plant mycosis and plant-parasitic nematode diseases [79].

## Natural Violacein Producers 

**Fig. 4:**
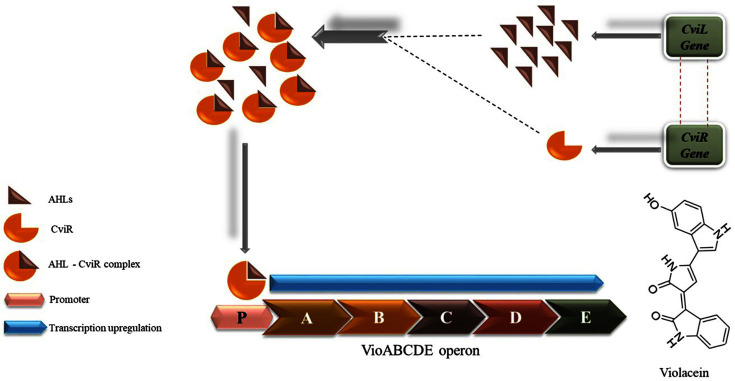
Quorum sensing in *C. violaceum*.

*C. violaceum* is a gram-negative, nonpathogenic bacterium that lives in soil and water, with a single circular chromosome of 4 × 10^6^ bp with an average G+C content of 64.8% [80]. Its optimal growth temperature is 30–35°C [81], and it can be grown in various media, including molasses, vegetable waste, food waste, etc. [82]. Native production of violacein in *C. violaceum* results in noticeable violet colonies on common laboratory media [81]. As an antibiotic with biocidal activity, its production is induced when the growth of C. violacieum enters the stationary growth phase or when nutrient supply is limited. This is a phenomenon broadly known as quorum sensing (QS), in which a class of signaling molecules (N-Acyl homoserine lactones, AHLs) participate in a feedback loop to regulate gene expression [83] (Fig. 4) [18]. This native producer has been engineered for improved violacein production. For example, *C. violaceum* strain ATCC 12472 and some mutants (CV12472, VIR24) show 25% enhanced violacein production by enhancing the production of AHLs that improved their QS behavior in the presence of sub-inhibitory concentrations of amikacin (1/4 MIC), erythromycin (1/8 MIC), gentamicin (1/2 MIC), kanamycin (1/6 MIC) and tetracycline (1/16 MIC) without involving the inhibition of bacterial growth [84].

Violacein is also produced by many *Janthinobacterium* strains, a genus of gram-negative soil bacteria with diverse energy metabolism abilities and tolerance to cold, ultraviolet radiation, and other environmental stressors. *Janthinobacterium* sp. B9-8 was first isolated from low-temperature sewage (5-10°C) in Xinjiang, China, having the ability to grow in a temperature range of 4–37°C. The production of violacein increased with increasing temperature till 25°C and then started decreasing. Hence, the B9-8 strain showed the fastest growth rate and violacein production ( 0.378 mmol/l) with the highest OD_600_ reaching 5.9 within 24 h at 25°C [85], as compared to other *Janthinobacterium* species and strains [82, 86, 87]. Recently, the genome sequence of B9-8 was reported, with 4.7 × 10^6^ bp and a G + C content of 48.72%. This genomic data reveals the gene cluster *vioABCDE* involved in violacein biosynthesis, which shows low query coverage (3–44%) and identity (66–87%) than the existing strains like *C. violaceum* (ATCC 12472), *J. lividum* (JCM 9043) and *J. lividum* (NBRC 12613) [85]. Another violacein-producing psychrotrophic XT1 strain (CGMCC 1194), identified as *J. lividum* XT1, was isolated from a glacier in China’s Xinjiang Province [86]. Violacein production by *J. lividum* XT1 was induced at a cultivation temperature lower than 20°C, with sucrose as carbon source, casein as nitrogen source, along with the addition of vitamins that made pigment productivity reach 10.19 mmol/l. The pigment content depended upon the temperature shift time, implying that low temperatures could really stimulate pigment formation for the XT1 strain. The cellular pigment content reached 2.33 mmol/dry cell weight, which was much higher than the previous reported (0.582 mmol/dry cell weight) in other *Janthinobacterium* strains [88].

Several strains belonging to *Duganella* sp. have also been identified with violacein-producing capacity. A total of seven strains from *Duganella* sp. are involved in violacein production [43]. A *Duganella* strain B2 was also isolated from Xinjiang, China [89], which produced 0.99 mmol/l of violacein in nutrient broth without any optimization. Under optimized culture condition with pH 6.71, potassium nitrate 1.18 g/l, beef extract 1.53 g/l, L-tryptophan 0.74 g/l, 25 ml medium in a 250 ml flask, with an inoculum size of 10% (v/v), it produced 4.71 mmol/l of violacein, a ~4.8-fold increase as compared to the normal conditions [19]. Another violacein-producing strain, *Duganella violaceinigra* str. NI28, was isolated from a temperate forest soil sample taken near Ulsan, South Korea, which is very close to the violacein producer *D. violaceinigra* YIM 31327 based on phylogenetic analysis. *D. violaceinigra* str. NI28 grew 25% faster on nutrient media and produced 45-fold more violacein as compared to its relative strain [43]. The much greater amount of crude violacein produced by this novel phenotypic variant was applied in the study of its biological activity against multidrug-resistant *S.aureus* [36]. Since *Duganella* is a mesophilic bacteria and most of its strains are isolated from places with a temperature range of 20-40°C, hence the difference in violacein production is not attributed to the temperature difference of the isolation source [36, 90]. Moreover, a recent study reported the isolation of a few more *Duganella* strains from a central Pennsylvania waterway which also showed the production of violacein [6].

*Collimonas* is a gram-negative, strictly aerobic bacterium that is isolated from acidic dune soils in the Netherlands [7]. The genus *Collimonas* was defined in 2004 for the first time [91]. The genetic sequence analysis revealed that *Collimonas* is closely related to *Herbaspirillum* and *Janthinobacterium* based on 16S rDNA sequences [7]. A new strain belonging to the genus *Collimonas*, designated as *Collimonas* CT isolated from the sea surface microlayer off the coast of Trøndelag, Norway, produced violacein with antibacterial properties [7]. Its growth rate increased at an incubation temperature of 25°C, without the inhibition of violacein production. *Collimonas* CT stopped producing violacein at 30°C and its growth stopped at 37°C [92]. *vioA* and *vioB* genes are involved in violacein biosynthesis and their fragments were amplified by PCR from the *Collimonas* CT genome and sequenced [93]. The idea of horizontal gene transfer was perceived by looking at the phylogenetic analysis of these sequences and their similarity with the biosynthetic gene cluster in *J. lividum* and *Duganella* sp. [7].

In genus *Massilia*, a few strains have been identified for the production of violacein. *Massilia* sp. BS-1 was a novel bacterium that had the ability to produce both violacein and deoxyviolacein. This bacterium contains 16S ribosomal DNA and showed 93% homology with *J. lividum*. A study showed that *Massilia* sp. strain B1 produced violacein provided with an exogenous supply of L-tryptophan and a small amount of L-histidine [94]. The amount of violacein in the presence of precursors of L-tryptophan (0-3.9 mM) and L-histidine (0.64 mM) turned out to be 1.3 mM in 10 ml of synthetic MM2 medium (0.2% glucose, 0.1% (NH_4_)_2_SO_4_, 0.4% Na_2_HPO_4_·7H_2_O, 0.2% KH_2_PO_4_, and 0.01% MgSO_4_·7H_2_O) [94]. *Massilia* sp. NR 4-1, another violacein-producing strain, was isolated and sequenced. A complete violacein biosynthesis pathway containing five genes *vioABCDE* was also explained. Its complete genome has 6,361,416 bp and a total of 5,285 coding sequences (CDSs) [8].

Like other gram-negative bacteria, violacein production is also reported in *Pseudoalteromonas luteoviolacea* sp. strains of 520P1 and 710P1 based on 16S ribosomal gene sequencing [95]. An upstream promoter element was found above gene cluster *vioA*-*vioE* which characteristically contained two continuous palindromic sequences that gave an insight into the link between the production of violacein and QS. Yi Wang and colleagues studied the signaling molecules involved in the production of violacein. They identified N-(3-oxooctanoyl)-homoserine lactone as the main molecule behind the production of violacein in the 520P1 strain [96]. Isolation of various strain samples from the Mediterranean led to the identification of a novel strain TC14 of *Pseudoalteromonas ulvae* which exhibited QS and production of violacein, by modulating AHLs involved in QS [9]. Previously, the discovery of one Luxl and five LuxR homologs in the 5.25 Mb genome of the 520P1 strain was reported [97]. Afterward, these Luxl and LuxR were characterized and named PalI and PalR1-R5. It was demonstrated that PalI was responsible for the production of two types of AHLs and thus is an AHL synthase, which is an essential component of the QS phenomenon, and the function of PalR1-R5 is yet to be elucidated [98].

Several violacein-producing microorganisms may turn out to be opportunistic pathogens and this factor is one of the drawbacks which lessens the benefits and applications of the violacein pigment [99]. Hence, the identification, isolation, and characterization of violacein from a non-pathogenic *Antarctic Iodobacter* strain is an important discovery. Several efficient methods for purification of the pigment generated 1.1 mg of violacein from 1 L of bacterial cells, giving a 0.01% yield of pure pigment [10]. The yield is not as much as has been reported in other bacterial species, but the discovery is still noteworthy, considering an *Antarctic Iodobacter* reporting the production of violacein for the first time. Moreover, the attributes of the *Antarctic Iodobacter* strain like non-pathogenicity and psychrotolerance make it valuable for pharmaceutical production of violacein and other potential biotechnological applications [10].

Furthermore, a mixture containing violacein and deoxyviolacein extracted from the psychrotrophic RT102 strain of was analyzed for its antibacterial activity. It showed growth inhibition and cell death of various bacteria like *S. aureus*, *P. aeruginosa*, *B. licheniformis*, *Bacillus megaterium*, and *Bacillus subtilis* at a concentration of 15 mg/l. Maximum violacein production was achieved by controlling various factors *i.e.* 10.77 mmol/l yield of violacein was obtained at 20°C at pH 6.0 and a dissolved oxygen concentration of 1 g/l [100]. In a recent study, P117 and P102 were isolated from freshwater Lake Winnipeg in Canada. P117 was related to *Massilia violaceinigra* (99.2%16S rDNA similarity) and P102 was related to *Janinthobacterium lividum* (99.3% 16S rDNA similarity). P117 produced a high concentration of deoxyviolacein whereas P102 produced a high concentration of violacein at pH 8.0 and 20°C, but higher deoxyviolacein was reported at 15°C in P117 [5].

All natural producers of violacein are summarized in Table 1.

### Recombinant Producers of Violacein

Since the whole pathway is encoded within the *vioABCDE* gene cluster, heterologous expression of this cluster in model industrial microorganisms (especially in L-tryptophan over-production chassis strains) enables further boosted production, mainly due to the faster growth, well-established culture procedures, and clear genetic background of these model organisms for efficient metabolic engineering. These microbial cell factories include *E. coli*, *C. glutamicum*, *Citrobacter freundii*, and *Enterobacter aerogenes*, as well as yeasts. The recombinant producers of violacein from recent reports are listed in Table 2.

*E. coli* is a well-established industrial bacterium which been employed as a cellular factory for the production of various metabolites and products [101]. Functional characterization and sequence analysis of violacein biosynthetic pathway in *E. coli* were traced back to 2000, using transposon mutagenesis as the tool to study *vio* gene cluster from *C. violaceum* [93]. Later, Brady *et al*. also functionally characterized the violacein pathway by introducing this pathway via an eDNA (environmental DNA) cosmid library in *E. coli* [102]. Violacein pathway was characterized via expression and purification of five VioA-E in *E. coli* and then in vitro production of violacein via these enzymes [29]. The stable reconstruction of the violacein biosynthetic pathway was done from *Duganella* sp. B2 in three different hosts, namely *E. coli*, *Citrobacter freundii*, and *Enterobacter aerogenes*. Among these three hosts, recombinant *C. freundii* strain produced the best results by giving out 4.893 mmol/l of crude violacein in shake flask cultures [15]. *E. coli* K12 strains were exploited and a stable hyper-producing violacein strain was made by introducing a series of changes to a range of strains by heterologous expression of *pPsx*-*vioABCDE*. The hyper-producing violacein strains (determined qualitatively via phenotype) were separated and checked for stable transmission of the plasmid across generations. Alongside this, a promoter mutation in the *pPsx*-*vio*-*ABCDE* was isolated which led to the over-production of violacein compared to the rest of the transformed *E. coli* strains used in this study [103]. Rodrigues *et al*. carried out systems-wide metabolic engineering of *E. coli* for enhanced production of violacein in a series of steps. First, the *vioABCE* cluster from *C. violaceum* was heterologously expressed in *E. coli* with an inducible AraC system, which led to the production of deoxyviolacein. Then, various bottlenecks in primary metabolism were identified to engineer for enhanced deoxyviolacein, including pathways in serine and aromatic amino acids biosynthesis, and non-oxidative pentose phosphate. Finally, *vioD* from *J. lividum* was introduced into the engineered strain and resulted in the production of extracted violacein 2.06 mmol/l of violacein with 99.98% purfity, under fed-batch conditions [104]. M. Y. Fang *et al*. reported high violacein yields without the exogenous addition of tryptophan by combining the upstream tryptophan pathway adjustment and the downstream violacein production pathway expression in *E. coli*. Two key genes in the tryptophan production pathway trpE^fbr^/trpD were overexpressed and competitive genes were knocked out (trpR/tnaA/pheA) (Fig. 1). Afterward, violacein biosynthetic pathway (pVio) was introduced and resulted in a titer of 5.09 mmol/l in the strain *E. coli* B2/Ped+pVio in a batch fermentation arrangement [21]. Later, the same group increased tryptophan production by fine-tuning of the TrpE^fbr^D, aroG^fbr^, and serA^fb^, yielding a titer of 1.31 mmol/l in flask culture [105]. VioE was found to be the rate-limiting enzyme and overexpression of VioE led to the highest production of violacein at 12.96 mmol/l without the exogenous addition of tryptophan [106]. Finally, the *E. coli* BL21 (DE3) with heterologous violacein pathway was screened in a high-throughput manner with the plasmid-based genome-scale sRNA library, which targets 1,858 *E. coli* genes. The gene *ytfR* (encoding sugar ABC transporter ATPase) was identified from the screen with the knocking down by sRNA resulted in a 600 % increase in violacein titer (2.024 mmol/l), as compared to the parent strain [107].

*C. glutamicum* is Generally Recognized as Safe (GRAS) and has a long history as a robust industrial workhorse for amino acids production [108]. Sun *et al*. introduced the vio gene cluster into the *C. glutamicum* ATCC 21850 strain, a tryptophan producer with a titer 0.471 mmol/l. By metabolic engineering and optimization of fermentation conditions, the violacein titer increased from 1.549 mmol/l to 15.727 mmol/l in a 3L bioreactor (Sun *et al*. 2016).

*Citrobacter freundii* is an infective bacterium and not an established cellular factory, but it shows a promising titer of violacein in fed-batch culture. A recombinant *C. freundii* by heterologous expression of *pCom10vio* plasmid was developed and a net titer of violacein at 12.02 mmol/l was obtained by maintaining optimal conditions of dissolved oxygen, pH, and providing a constant supply of glycerol, NH_4_Cl, and L-tryptophan at 16 ml/h (Yang *et al*. 2011).

Moreover, *Vibrio natriegens* is a non-pathogenic bacterium that possesses lots of special benefits such as fast growth, tolerance of high salt concentration, and so on [109]. *V. natriegens* having the ability to encode tryptophan was integrated with *pVio* plasmid containing five enzymes (*vioABCDE*) and cultured in both minimal media with different carbon sources and LBv2 rich medium and tested for the production of violacein and deoxyviolacein [110].

*Saccharomyces cerevisiae*, a eukaryotic model organism, is also a well-established cellular factory and has been employed for the production of many industrially important metabolites and alcohols [111]. This gene cluster *vioABCDE* has been expressed in *S. cerevisiae* in several studies, but the violacein produced was not economically feasible as compared to the bacterial systems. Instead, researchers took advantage of the vivid colors from violacein and intermediates in its biosynthetic pathways to evaluate their designs in the field of synthetic biology, which we address in the following section.

Another yeast species, *Y. lipolytica*, has proved itself as an efficient cellular factory for the production of many chemicals including both lipid and non-lipid substances [112], which also has the GRAS status by the FDA for the production of many commercial compounds. Wong *et al*. developed YaliBrick, an assembly method compatible with BioBricks standard, to tune gene expression of complex metabolic pathways in *Y. lipolytica*. The violacein biosynthetic pathway was tested by this method in *Y. lipolytica* with resultant clones of different purple colors [23]. Subsequent optimization of the culture conditions and extraction methods resulted in 0.203 mmol/l violacein and 0.0161 mmol/l deoxyviolacein in the presence of ethyl acetate as an extraction solvent [113]. As the precursor for violacein biosynthesis is L-tryptophan, in a later study, the rate-limiting steps of the shikimate pathway for aromatic amino acids synthesis were debottlenecked in Y. Lipolytica. They resulted in enhanced de novo synthesis of violacein, reaching the highest titer of 1.065 mmol/l [24]. Meanwhile, another group worked on the downstream processes for the extraction of violacein from *Y. lipolytica*. They developed a temperature-sensitive system that utilized sodium dodecyl sulfate surfactant as well as tetrabutylammonium chloride salt in an integrated solid-liquid extraction of violacein, and its subsequent purification via cloud point separation. Their results for violacein extraction indicated that violacein and protein extraction can be tuned by changes in the [N_4444_]Cl–SDS molar ratio, a tunability not available for solutions solely containing surfactants. Furthermore, the violacein extraction selectivity using [N_4444_]Cl–SDS is notably superior to that achieved using the commercial nonionic surfactant Triton X-114 [114].

### Applications in Synthetic Biology

In recent years, researchers in synthetic biology and metabolic engineering have frequently utilized the violacein pathway to evaluate their designs. This is mainly due to the easily detectable hues of violacein and the intermediates on the pathway as the reporters, as well as the proper number of genes in the cluster, similar to other systems metabolic engineers typically meet to manipulate. The applications of violacein in synthetic biology are summarized in Table 4.

**Fig. 5:**
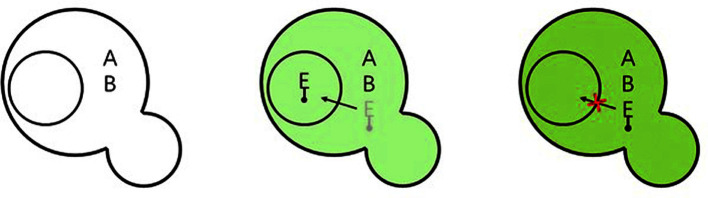
IPA imine dimer is colorless, so the yeast is also colorless when *VioA* and *VioB* are only freely expressed in the cytosol (left). The optimized tag ePTS1 was fused to *VioE*, to sequestrate it in the peroxisome, and to reduce PDV production (middle). However, when the PTS1 import is deficient by using a pex5Δ strain, there is a higher PDV production.

In the applications as reporters, the Dueber group fully characterized yeast peroxisome for pathway compartmentalization to improve the biocatalytic efficiency by using *vioABE*, whose product prodeoxyviolacein (PDV) or its derivative is visibly green and highly red fluorescent (Fig. 4). By expressing *vioA* and *vioB* in cytosol and screening signal peptide library fused to C-terminus of *vioE* for peroxisomal translocation, they identified an enhanced combined linker-PTS1 tag (LGRGRR-SKL), which yielded a 95% reduction of PDV compared with a 63% reduction with the canonical PTS1 tag (SKL). Then, the optimized tag ePTS1 was fused to β-glucosidase, *vioA*, *B*, or *E*, respectively, to estimate the permeability of the peroxisomal membrane, which indicates the membrane has a cut-off limit of small molecules with molecular weight between 571-733 Da (Fig. 5). They also experimentally proved VioA’s substrate (tryptophan) and product (IPA imine) can freely cross while IPA imine dimer cannot [26]. Such a well-characterized translocation system was then applied in *S. cerevisae* to sequester the toxic truncated norcoclaurine synthase (tNCS), from *Coptis japonica*, which catalyzes the first committed step for the biosynthesis of benzylisoquinoline alkaloids (BIAs). They further identified peroxisome protein capacity as a limiting factor and induction of larger peroxisomes was achieved by the addition of oleate, leading to further improvement of BIA production [115].

Synthetic biologists favor the use of the violacein biosynthetic pathway also due to the proper number of five genes in the cluster. For example, the violacein pathway has been used as the reporter to evaluate the efficiency of gene assembly toolkits developed based on Golden Gate assembly in *E. coli* [116] and yeast [117]. Lee *et al*. constructed the combinatorial library of violacein pathway genes in *S. cerevisiae* with different promoter strengths, and applied and trained a linear regression model. This model enabled the prediction of optimized production based on a few measurements only comprising 3% of the total library, which should also be useful in engineering new pathways in other systems [25]. Similarly, an efficient machine-learning workflow in combination with YeastFab Assembly strategy (MiYA) was developed that could optimize the large biosynthetic genotypic space of heterologous metabolic pathways in *S. cerevisiae*. From the initial screen of 24 random strains, this method allowed identification of a combination with 2.42-fold improved violacein production among 3125 possible designs. Furthermore, this method also reliably predicted the synthesis of very pure violacein, avoiding the branch pathway for the by-product deoxyviolacein formation [118]. In another study, the beneficial effect of metabolite channeling was tested by using Artificial Protein Scaffolds (AProSS). AProSS brings VioC, VioD, and VioE in proximity, avoiding diffusion of intermediates, so that violacein and deoxyviolacein titers were increased by 29% and 63%, respectively [119]. In another such experiment, a bistable switch was employed in *S. cerevisiae* through a toolkit having specialized transcription factors and promoters that are designed to control the expression of *vioC* and *vioD* genes in the violacein biosynthetic pathway. These DNA parts with efficient performances can lead to the development of fine genetic circuits, that can be utilized in several biotechnological applications [120].

In metabolic pathway engineering, the vivid violet hue of violacein or deoxyviolacein has been utilized to evaluate genetic mutants for improved production by high-throughput ways. For example, Jones *et al*. constructed a T7 promoter mutant library in *E. coli* by site-directed mutagenesis, from which promoters of different strengths were obtained. The combinatorial library of *vioA*, *B, C, D*, and *E* was then built up using these promoters, and violacein production can be directly evaluated in a high-throughput manner, based on the violet color. Such fine-tuning method for multiple genes, called ePathOptimize, can be straightforwardly applied to other multi-step pathways to balance metabolic flux [121]. In another study, the adaptive laboratory evolution (ALE) technique was employed to increase violacein production utilizing galactose as a carbon source. A tryptophan-responsive biosensor was used to apply selection pressure on tryptophan-producing cells during this technique. An evolved population of cells capable of effectively catabolizing galactose to tryptophan from the biosensor-assisted ALE was obtained. The population was then used to choose the best violacein producer [122].

Finally, the accumulation of heavy metals in the environment including water bodies, soil, and foods caused by various anthropogenic activities of humans has become a matter of concern with increasing risk factors and morbidity [123]. Genetic engineering of microorganisms to gain quantitative fluorescent or enzymatic signals in response to heavy metals exposure has a great potential for the assessment of the bioavailability of heavy metals in the environment [124]. Metabolism of natural pigments *e.g.*, lycopene, carotenoid, pyocyanin as reported have been used to derive whole-cell biosensors that give visible output signals in response to environmental pollutants [125]. A whole-cell customized biosensor was designed by constructing violacein pathway under the control of Pb (II) -dependent metalloregulator PbrR. In response to lead exposure, lead-sensitive PbrR regulator stimulated intracellular violacein production which was quantified by colorimetric method at 490 nm. This violacein-based biosensor is highly sensitive and can detect Pb (II) concentration as low as 0.1875 μmol/litre [27]. Another violacein-based bioavailable Hg(II) biosensor was constructed under the control of mercury resistance (mer) promoter and mercury resistance regulator (MerR). The biosensor cells responded to Hg(II) preferentially, and the response was unaffected by other interfering metal ions. In a colorimetric technique, the exponentially increasing violacein-based biosensor identified concentrations as low as 0.39 μmol/litre Hg(II), with a linear relationship seen in the concentration ranging from of 0.78 to 12.5 mol/l. In a colorimetric approach and a Hg(II)-containing plate sensitivity test, non-growing biosensor cells responded to concentrations as low as 0.006 mol/l Hg(II), and the linear connection was shown in a very limited concentration range. These results showed that the violacein-based biosensor could be used to assess the ecotoxicity of environmental water samples having mercury pollutants [126].

## Conclusion

This review focuses on the bacterial secondary metabolite, violacein, which has shown promising potential in various biotechnological applications. In this compilation of recent research on violacein, we have demonstrated the increased interest in natural and recombinant producers of violacein. Similarly, there is a lot of interest in its various properties of industrial, biological, and research significance. The structure and synthetic biology perspective of violacein has also been discussed. Therefore, there is wide room for future research in increasing its yield, fermentation, and applications.

## Figures and Tables

**Table 1:** Natural producers of violacein.

Strain	Origin/source	Characteristics	Yield	References
*C. violaceum*	Soil and water	Facultative anaerobe	0.43 g/l	[79, 125]
*Janthinobacterium* sp. B9-8	Xinjiang, China	Low-temperature sewage(5–10°C), 98.6% similarity with that of *J. lividum*.	130 mg/l	[85]
*J. lividum*	Glacier and on the skin of amphibians	Psychrotrophic, kills *Batrachochytrium dendrobatidis*, biofilm development, antibacterial properties against *E. coli*, *Staphylococcus aureus*, and the *S. aureus* MRSA, antifungal against *Candida albicans*, *C. parapsilosis*, and *C. krusei*, synthesis of antimicrobial polyamide fabrics, immediate dying.	1.828 g/l	[84] [77] [126]
*J. lividum* XT1	Xinjiang, China	Violacein production in the presence of sucrose, casein vitamins, and minerals at > 20°C.	3.5 g/l	[84]
*Duganella* B2	Xingjiang, China	Plackett–Burman and Box–Behnken More violacein production than *C. violaceum* under optimum conditions.	1.62 g/l	[19]
*Duganella violaceinigra* str. NI28	Near Ulsan, South Korea	Relative of *Duganella violaceinigra* YIM 31327 produced (45-folds more violacein than *D. violaceinigra* YIM 31327 effective against multidrugresistant *Staphylococcus aureus*.	18.9mg/l	[43] [12]
*Collimonas* CT	The coast of Trøndelag, Norway	Closely related to *J. lividum* and *Duganella* sp. B2, horizontal gene transfers maximum pigment production at 20–25°C, antimicrobial activity against Micrococcus luteus (ATCC 9341).		[7]
*Collimonas* fungivorans gen.	Hyphae of several soil fungi	Most closely related genera are *Herbaspirillum* and *Janthinobacterium*. Highest growth rates at 20–30°C.		[91] [7]
*Massilia* sp. BS-1	Soil	93% homology with *J. lividum*, utilizes tryptophan and L-histidine for violacein production.	0.446 g/ 10 ml	[94]
*Massilia* sp. NR 4	Topsoil under nutmeg tree, Torreya nucifera in Korean national monument, Bijarim Forest	Aerobic, non-spore-forming rod-shaped, *Massilia* colonization on the seed coat, radicle, or roots protect against infection by soil-borne plant pathogen Pythium aphanidermatum at a plant developmental stage.		[8][129]
*Pseudoalteromonas* sp. (Strains 520P1,710P1)	Coast of Japan	Research provided a deep insight into the phenomenon of quorum sensing in these strains.		[95][98]
*Pseudoalteromonas* sp. (TC14)	Mediterranean	A novel strain exhibited quorum sensing.		[9]
*Antarctic Iodobacter*	Antarctic territory	Non-pathogenic genus, psychrotolerant, a member of the family Oxalobacteraceae.	1.1 mg/l	[10]
Antarctic bacterial isolate		Highest yield at 20°C in Tryptic Soy Broth medium supplemented with 3.6 g/l glucose, double yield in a 5 L bioreactor.	77 mg/l	[66]
Psychrotrophic bacterium RT102		Antiproliferative activity, growth inhibition, and cell death of *Staphylococcus aureus*, *Pseudomonas aeruginosa*, *Bacillus licheniformis*, *Bacillus megaterium*, and *Bacillus subtilis*.	3.7g/l	[128]
Psychrotrophic bacterium P117 and P102	Freshwater, Lake Winnipeg	P117 is related to *Massilia violaceinigra*, which produces a higher concentration of deoxyviolacein and P102 is related to *Janinthobacterium*. produces a higher concentration of violacein.		[129]

**Table 2:** Recombinant producers of violacein.

Recombinant strains	Metabolic engineering	Titer	Reference
*Citrobacter freundii*	Heterologous expression of pCom10vio plasmid in *C. freundii*.	4.13 g/liter	[20]
*Corynebacterium glutamicum*	*Corynebacterium glutamicum* ATCC 21850 strain employed for violacein production in a 3L bioreactor.	5.436 g/l	[22]
*E. coli*	VioABCE cluster from *C. violaceum* was expressed in *E. coli* *dVioL* under an inducible ara C system. Metabolic engineering of serine, non-oxidative pentose phosphate, chorismite, and tryptophan biosynthesis pathways and integration of *VioD* from *J. lividum* produced violacein.	0.710 g/l	[102]
	Up-regulation of endogenous tryptophan pathway by overexpression of trpE^fbr^/trpD,knockoutofcompetitivegenes(trpR/tnaA/pheA)anddownstreamintegrationofviolaceinbiosyntheticpathwayin *E. coli* B2/Ped +pVio resulted in violacein production.	1.75 g/l	[21]
	Introduction of *vioABCDE* gene cluster in B8/TRPH1 strain engineered to accumulate tryptophan from glucose showed that *VioE* is a rate-limiting enzyme and its increased concentration gave off an increased amount of violacein.	4.45 g/l	[104]
	Introduction of expanded sRNA expression vector pColA-Sm R harboring genome-scale sRNA library in *E. coli* BL21 (DE3) and knocking down of ytfR gene by sRNA gave good violacein production.	0.695 g/l	[105]
*Yarrowia lipolytica*	Violacein production by the integration *vioABCDE* gene cluster in DNA assembly named YaliBrick in *Y. lipolytica*.	-	[23]
	De novo synthesis of violacein in *Y. lipolytica* by eliminating ratelimiting step.	0.366 g/l	[24]

**Table 3:** Biological activities of violacein.

	Biological Properties	References
Anti-microbial activity	Violacein from *Janinthobacterium* sp. inhibits Multi-Drug Resistant bacteria *P. aeruginosa* and *S. marcescens*.	[42]
	Violacein against *S. aureus* ATCC 29213 and *S. aureus*, ATCC 43300 showed four times greater activity as compared to vancomycin.	[13]
	Violacein integrated silk fabric against *S. aureus* caused bacterial population reduction of 81.25%.	[40]
	Violacein loaded in poly-(D, L-lactide-co-glycoside) nanoparticles showed three times more antibacterial activity as compared to free violacein against *S. aureus* ATCC 25923.	[38]
	Violacein from cutaneous bacteria of amphibians showed antifungal activities against *Batrachochytrium dendrobatidis* and *Batrachochytrium salamandrivorans*.	[47]
	Antifungal activities against *Botrytis cinerea*, and *Colletotrichum acutatum*, *Colletotrichum glycines*, *Colletotrichum orbiculare*, *Gibberella zeae*, *Phytophthora capsica*, and *Verticillium dahlia*, etc.	[48] [41]
	Inhibitory effects against viruses like Simian rotavirus SA11, HSV-1, and Poliovirus type 2.	[49]
	Anti-malarial activity against *Plasmodium falciparum* and *Plasmodium chabaudi* in mice.	[50]
Anti-parasitic activity	Anti-trypanosomal activities against *Trypanosoma cruzi*.	[130]
	Mild cytotoxic activity against *Trypanosoma brucei gambiense*.	[51]
	Antinematodal activity against *C. elegans*.	[53]
	Anti-leishmanial activity against *Leishmania amazonensis*.	[52]
	Violacein administered orally for 4 days at a dose of 40 mg/kg showed immunosuppressive activity against delayed-type hypersensitivity caused by sheep red blood cells.	[55]
Immunomodulatory activity	Modulation of central and peripheral antinociceptive activities.	[55]
	Violacein against HeLa (cervix cell carcinoma) cell lines rendered them sensitive to cisplatin (an anti-tumor drug).	[66]
Anti-cancerous activity	Violacein encapsulated with Pectin-Gelatin showed anti-cancerous activity on HTC-116 colon cancer cell lines.	[67]
	Violacein downregulates CXCL12/CXCR4 interaction in breast cancer cell lines MCF7.	[131]
Nephroprotective activity	Violacein and silver nanoparticles making a dyad system, to structurally bind and inhibit TFAM at the interface of the TFAM-DNA complex against cancer proliferation.	[68]
	Violacein showed nephroprotective activity against heavy metals and gentamicin through the antioxidant property.	[69]
Anti-diarrhoeal and ulcer-protective property	Violacein showed ulcer-protective characteristics against ethanol-induced ulceration, with anti-ulcer activity peaking at 40 mg/kg. Violacein (40 mg/kg) inhibited castor oil-induced diarrhoea in rats by 87.84 percent.	[70]

**Table 4:** Applications of violacein in synthetic biology.

Applications		Reference
Reporters	Improvement of the biocatalytic efficiency of *vioABE* having prodeoxyviolacein (PDV) or its derivative, with visible green and red fluorescence. Estimation of the permeability of peroxisomal membrane (up to the molecular weight between 571-733 Da) by the fusion of optimized tag ePTS1 to β-glucosidase, VioA, B, or E.	[26]
Benzylisoquinoline alkaloids (BIAs)	Application of a well-characterized translocation system for the catalysis of the first step for the biosynthesis of BIAs from *Coptis japonica* in *S. cerevisae* to sequester tNCS. Induction of larger peroxisomes by addition of oleate to improve BIA production.	[113]
T7 Promoter mutant library	VioA, B, C, D, and E libraries were built up using high-strength promoters through which the violacein production can be directly evaluated. Such fine-tuning method for multiple genes called ePathOptimize balances the metabolic flux.	[119]
Golden Gate assembly	Evaluation of the efficiency of gene assembly toolkits developed via Golden Gate assembly in *E. coli* and yeast.	[114, 115]
Biosensors	The construction of a highly sensitive whole-cell biosensor that can detect Pb (II) concentration as low as 0.1875 μmol, was designed under the control of T7 lac promoter in *E. coli*.	[27]
	Hg(II) biosensor controlled by mer promoter and MerR regulator to assess the ecotoxicity of environmental water samples having mercury pollutants	[124]
Combinatorial libraries	Combinatorial libraries for matching promoters with the adjacent suitable genes and characterization of constitutive promoters for maximum titer production of violacein.	[25]
Artificial Protein Scaffolds (AProSS)	VioC, VioD, and VioE were brought in proximity via Artificial Protein Scaffolds (AProSS) increased the yield of violacein and deoxyviolacein by 29% and 63% respectively.	[117]
Bistable switch	Application of a Bistable switch in *Saccharomyces cerevisiae* for controlling *vioC* and *vioD* which switches on alternative *vioC* and *vioD* pathways on demand.	[118]
YeastFab Assembly strategy (MiYA)	MiYA was developed in *S. cerevisiae* and could allow the identification of a combination with 2.42-fold improved violacein production among 3125 possible designs and predict the synthesis of pure violacein, avoiding the branch pathway.	[116]
